# Increasing sperm production and improving cryosurvival of semen in aged Thai native roosters as affected by selenium supplementation

**DOI:** 10.5713/ab.23.0079

**Published:** 2023-06-26

**Authors:** Supakorn Authaida, Ruthaiporn Ratchamak, Wuttigrai Boonkum, Vibuntita Chankitisakul

**Affiliations:** 1 Department of Animal Science, Faculty of Agricultural, Khon Kaen University, Khon Kaen 40002, Thailand; 2 The Research and Development Network Center of Animal Breeding and Omics, Khon Kaen University, Khon Kaen 40002, Thailand

**Keywords:** Aging Rooster, Fertility, Frozen Semen, Lipid Peroxidation, Malondialdehyde (MDA)

## Abstract

**Objective:**

Aging roosters typically exhibit subfertility with decreasing semen quality, furthermore Thai native roosters reared in rural areas are raised for a longer duration than their usual lifespan. The present study therefore aimed to assess the effect of selenium supplementation as an antioxidative substance in diets to improve the semen cryopreservation of aged roosters.

**Methods:**

Semen samples were collected from young (n = 20) and aged (n = 20) Thai native roosters (Pradu Hang Dum) at 36 and 105 weeks of age when starting the experiment, respectively. They were fed diets either non-supplemented or supplemented with selenium (0.75 ppm). Fresh semen quality and lipid peroxidation of fresh semen was evaluated before cryopreservation using the traditional liquid nitrogen vapor method. Post-thaw sperm quality and fertility potential were determined.

**Results:**

Advancing age is unrelated to decreasing fresh semen quality (p>0.05). However, lipid peroxidation in rooster semen depended on age, and the malondialdehyde (MDA) concentration increased in aged roosters (p<0.05). Selenium supplementation in diets significantly decreased the MDA concentration and increased the sperm concentration (p<0.05). In contrast, cryopreserved semen was affected by advancing rooster age, and selenium influenced sperm quality (p<0.05). Younger roosters had higher post-thaw sperm quality and fertility potential than aged roosters (p<0.05). Likewise, diet selenium supplements improved post-thaw sperm quality and fertility compared with the non-supplement group.

**Conclusion:**

Rooster’s age does not influence the rooster sperm quality of fresh semen, while sperm cryotolerance and fertility were greater in young roosters than in aged roosters. However, sperm of aged roosters could be improved by dietary selenium supplementation.

## INTRODUCTION

Traditionally, native Thai chickens have been raised mainly for home consumption and play an essential role in the economy at the community level. These chickens are typically raised on backyard farms for longer utilization by small farmers with poor husbandry practices, and insufficient nutrition often results in poor growth performance [1], low egg production [2], and low fertility [3]. However, large-scale commercial rearing of exotic chicken breeds subjected to efficient meat and egg performances has become a large part of the supply chain, decreasing the number of Thai native chickens and their genetic diversity. One of the strategies to conserve the genetic diversity of those purebred, semen cryopreservation is a preferable procedure. However, the ability to freeze and thaw sperm depends on several factors, including ejaculated semen quality and cryotolerance during semen cryopreservation.

Previous studies have demonstrated a negative association between ejaculated semen quality and increasing age [4]. The effects of aging on cellular and hormonal changes, such as falling testis weight and decreased testosterone concentration leading to sperm quantity and quality of fresh semen, are decreased [5], subsequently affecting potential fertilization quality [6,7]. The maximum semen volume and sperm concentration were reported at different ages according to rooster breeds [8]. For instance, the maximum obtained from roosters of 24 to 48 weeks of age then gradually decreased in White Leghorn roosters [9]. Testicular functional integrity is achieved throughout rooster age (30 to 50 weeks). At the same time, there was a noticeable reduction in the amount of sperm in the tubules, mild atrophy, sperm degeneration, and calcification of the seminiferous tubules at 60 weeks of age. These changes become more noticeable as the rooster ages [5]. Escorcia et al [10] showed that oxidative damage was more significant in the testicles of roosters between 36 and 72 weeks of age than in those at 30 weeks of age. It might be inferred that the quality of reproductive capacity would begin to decline as the rooster ages. Therefore, in almost all commercial flocks, roosters are commonly used for breeding for 36 weeks or up to 55 weeks of age [5]. In contrast, native Thai roosters in rural backyard farms are raised longer than their usual lifespan [11]. Therefore, conserving sperm from aging roosters is possible. However, rooster age had a detrimental effect on post-thaw sperm function [12].

Another crucial factor that affects cryopreserved semen is the high cryoinjury of sperm cells, which mainly results from cold shock and oxidative stress during the freezing procedure, leading to membrane damage and sperm. High amounts of polyunsaturated fatty acids (PUFAs) in the chicken plasma membrane increase their susceptibility to oxidative stress. The antioxidant system is therefore required to protect sperm membranes against peroxidative damage. However, the natural antioxidant contents were apparently affected by semen processing and cryopreservation. Increasing membrane fluidity [13] or supplementing several antioxidant substances [14] are therefore introduced to improve the quality of frozen rooster semen.

Selenium, an essential element for chickens, is an antioxidant that defends cells from free radicals. A severe selenium deficit is linked to lower poultry development and reproduction [15]. Selenium protects cells in a cell culture system from oxidative damage by reducing free radical generation and inhibiting lipid peroxidation [16]. At 0.5 ppm, selenium supplementation boosted the enzyme glutathione peroxidase activity, lowered blood malondialdehyde (MDA) levels, and increased sperm volume [17]. Even though many research publications used selenium to improve chicken semen production; however, only one study reported the effect of selenium supplementation on cryopreserved rooster semen. According to Chauychu-noo et al [18], the optimal concentration of selenium supplemented on a diet between 0 and 0.9 ppm was examined. The researchers suggested that selenium supplementation in diets at 0.6 ppm was recommended to improve the quantity and quality of fresh and frozen semen. Within those levels, the toxicity or adverse affected on semen quality were not found. However, that research did not mention the factor of rooster age, which is a main factor in the present study, as mentioned above.

To our knowledge, limited studies on semen cryopreservation have been conducted on roosters of advanced age. Additionally, there have never been reports on oxidative stress in the fresh semen of Thai native roosters. In the present study, it is important to evaluate the differences in oxidative stress in roosters of different ages before using antioxidant supplementation (selenium) in diets to improve the semen cryopreservation of aged roosters. Different stages of sexual maturity (young and aged roosters) were included in the study. This finding helps extend the reproductive life of aged roosters and maintain semen cryopreservation to conserve genetic diversity in aging Thai native chickens.

## MATERIALS AND METHODS

The Institutional Animal Care and Use Committee approved the experimental procedures based on the Ethics of Animal Experimentation of the National Research Council of Thailand (record no. IACUC-KKU-90/65; reference no. 660201.2.11/ 632 (114)). Unless otherwise stated, all chemicals used in this study were purchased from Sigma-Aldrich Chemical Co. (St. Louis, MO, USA).

### Experimental animals

The roosters were composed of young (n = 20) and aged (n = 20) Thai native roosters (Pradu Hang Dum) at 36 and 105 weeks of age when starting the experiment, respectively. The roosters were kept in individual cages in an open-house system with an average temperature of 27°C and relative humidity of 72%. Each rooster was fed 130 g of commercial diet daily, providing 16% crude protein and a base of selenium of 0.15 ppm, as determined by Central Laboratory (Thailand) Co. Ltd. The water was given *ad libitum* throughout the experimental period.

Forty-eight Thai synthetic hens (Kaen Thong breed) at 32 weeks of age with egg production >80% were used for the fertility test. The hens were housed individually, fed approximately 110 g of commercial diet daily, and given water *ad libitum*.

### Experimental design

Based on a previous report on rooster semen cryopreservation [18], the concentration of the selenium supplementation was 0.6 ppm. To examine the effect of selenium supplementation in diets on semen cryopreservation of aged roosters, the roosters were fed a basal diet supplemented with or without 0.6 ppm selenium (ECONOMASE TM Alltech, Inc., Nicholasville, KY, USA) for at least 14 days before starting semen collection and continued supplementing until the end of the experiment. The total level of selenium in the experiment group is 0.75 ppm.

The semen samples were collected twice a week. Fresh and frozen semen quality was evaluated. Additionally, the fertilizing ability of the frozen–thawed semen was tested. The experiment was repeated six times.

### Semen collection

The dorso-abdominal massage technique was used to collect sperm twice a week. Individual rooster semen was collected in 1.5 mL microtubes with 0.1 mL of Schramm diluent, which was composed of 0.7 g magnesium acetate, 28.5 g sodium glutamate, 5 g glucose, 2.5 g inositol, and 5 g potassium acetate, all dissolved in 1,000 mL of deionized water; the pH was 7.1, and the osmotic pressure was 395 mOsm/kg. To maximize semen quantity and quality, the same person conducted the collection simultaneously under the same conditions. Great care was taken to avoid contamination of the sperm with feces, urates, and clear fluid, all of which degraded the sperm quality. Within 20 min of collection, sperm samples were transferred to the laboratory, and semen quality was determined. Semen with the following criteria were pooled to eliminate the individual effects for further semen cryopreservation. The criteria were as follows: semen volume, at or above 0.3 mL; sperm concentration, ≥3×10^9^ spz/mL; and motility (MOT) score, ≥4.

### Evaluation of fresh sperm quality

The volume of ejaculated semen was measured by aspirating the whole semen sample into a 1 mL syringe with an accuracy of 0.02 mL.

The sperm concentration was determined using a hemocytometer chamber. One microliter of semen sample was diluted with 999 μL (1:1,000) of 4% sodium chloride. A drop of semen sample was placed on a hemocytometer, and the reading was recorded under a compound microscope (400× magnification). The sperm concentration was expressed as billions (10^9^) of sperm cells/mL.

The intensity of the waves formed by sperm movements was assessed. A drop of 10 μL semen was placed on a slide without a coverslip, examined under a compound microscope (100×), and scored on a scale of 0 to 5 (0 = no sperm movement; 5 = very rapid waves and whirlwinds visible, with more than 90% of sperm showing a forward movement).

The MDA concentration as an index of lipid peroxidation in the semen samples was measured using the thiobarbituric acid reaction as previously described [13]. Semen samples from each treatment were adjusted to 250×10^6^ spz/mL; then the semen was added to 0.25 mL ferrous sulfate (0.2 Mm; Riedel-de haen, 12354 Seelze, Germany) and 0.25 mL ascorbic acid (1 mM; Sigma, A5960 Steinheim, Germany) and incubated at 37°C for 60 min, after which 1 mL trichloroacetic acid (15%; Sigma, T6399, Germany) and 1 mL thiobarbituric acid (0.375%; Sigma, T550, Germany) were added and boiled in water for 10 min, after which the samples were cooled to 4°C to stop the reaction. Finally, the samples were centrifuged at a controlled temperature of 4°C at 4,000× g for 10 min and analyzed using UV-vis spectrophotometry (Analytikjena Model Specord 250 plus; Analytikjena, Jena, Germany) at 532 nm.

### Semen cryopreservation

After fresh semen evaluation, pooled semen was diluted 1:3 (v:v) in Schramm diluent and cooled to 5°C for 1 hour (1°C per 3 minutes). Afterward, DMF (N, N-dimethylformamide) was added to a semen extender at a final concentration of 6% (v/v) and mixed. Semen was loaded into 0.5 mL plastic straws and immediately closed with polyvinylpyrrolidone powder. The final sperm concentration in each 0.5 straw was approximately 625×10^6^ spz/mL. After 15 min of equilibration, the filled straws were placed horizontally on a rack above the liquid nitrogen surface at −35°C for 12 min, placed at −135°C for 5 min, and finally plunged into liquid nitrogen for storage at −196°C until analysis. Thawing was achieved at 5°C for 5 min in cool water. The freezing and thawing protocols were conducted according to our previous study [13].

### Evaluation of frozen sperm quality

Total MOT and progressive motility (PMOT) were analyzed using a computer-assisted sperm analysis system (version 10 HIM-IVOS; Hamilton Thorne Biosciences, Beverly, MA USA). For each sample, two slides (maintained at 25°C) were filled with 5 μL diluted semen, and three fields per slide were recorded for 10 s using a 10× phase-contrast objective (Olympus, Tokyo, Japan) in conjunction with a digital camera (Olympus DP 71/25). They were operating at 30 frames per second (60 Hz). Sperm was defined as nonmotile when the average path velocity was less than 5 μm/s, and sperm was considered progressively motile when the average path velocity was greater than 20 μm/s, and the straightness index was 80%.

The percentage sperm viability was determined by SYBR-14 and propidium iodide (PI) (LIVE/DEAD Sperm Viability Kit; Invitrogen TM, Thermo Fisher Scientific, Waltham, MA, USA), as described by Partyka et al [19] with minor modifications. Briefly, the semen sample was first incubated for 10 min with 5 μL of SYBR-14 solution, followed by staining with 5 μL PI for 5 min. The cells were then fixed with 10% formaldehyde. For assessment, at least 200 sperm cells were analyzed under an IX71 fluorescence microscope (Olympus, Japan) at 400× magnification. PI-negative cells showing red fluorescence were dead, and SYBR-14-positive cells showing green fluorescence were considered to be live, with sperm plasma membrane intact.

The MDA content was determined using 200 μL (250×10^6^ spz/mL) frozen-thawed semen. The protocol was conducted in a similar manner with fresh semen samples.

### Fertility

Forty-eight hens were assigned randomly to four groups (twelve hens/group) with six replications. All hens were inseminated with 0.4 mL (500×10^6^ spz) of frozen-thawed semen once a week. Insemination was performed between 15.00 and 17.00 h. Eggs were obtained between days 2 and 8 following inseminations. The total number of eggs collected from the hens in each treatment group was recorded. Candling the eggs on day 7 of incubation determined fertility.

### Statistical analysis

The study used a factorial experiment in a completely randomized design with two factors monitored at two levels and six replications for all parameters. Factor A was the rooster age (young and aged), and factor B was the selenium supplement in diets (presence-absence of selenium). The effects of rooster age, selenium supplement in diets, and their interaction mean values for each parameter were compared using Tukey’s post hoc test (*F-value* and p<0.05 were presented). All data were arcsine transformed before statistical analysis, and results are expressed as mean values±standard error.

## RESULTS

### Determination of fresh semen quality and lipid peroxidation

The interaction effect between rooster age and selenium was nonsignificant for all quality parameters of fresh semen with *F-values* of 0.11, 0.25, and 0.38 (p>0.05; Table 1) for semen volume, sperm concentration, and mass movement, respectively. The age of the rooster did not influence any semen quality parameter with *F-values* of 1.34, 0.29, and 0.34 (p> 0.05), respectively, while only the effect of selenium was significant for the sperm concentration with an *F-value* of 15.96 (p<0.01). For lipid peroxidation, the interaction effect between rooster age and selenium was nonsignificant for MDA levels of fresh semen with an *F-value* of 0.28 (p>0.05). However, aged roosters had higher MDA levels than young roosters, with an *F-value* of 16.35 (p<0.01). Additionally, selenium supplementation in diets significantly decreased the MDA concentration with an *F-value* of 17.53 (p<0.01).

### Determination of cryopreserved semen quality and fertility

The levels of significance for the influence of rooster age and selenium on sperm MOT, sperm viability, and lipid peroxidation of cryopreserved semen are demonstrated in Table 2. The interaction effect between rooster age and selenium was significant for sperm viability and MDA level with *F-values* of 10.56 and 6.54, respectively (p<0.05), but insignificant for total MOT and PMOT with *F-values* of 1.72 and 1.49, respectively (p>0.05).

**Figure 1:**
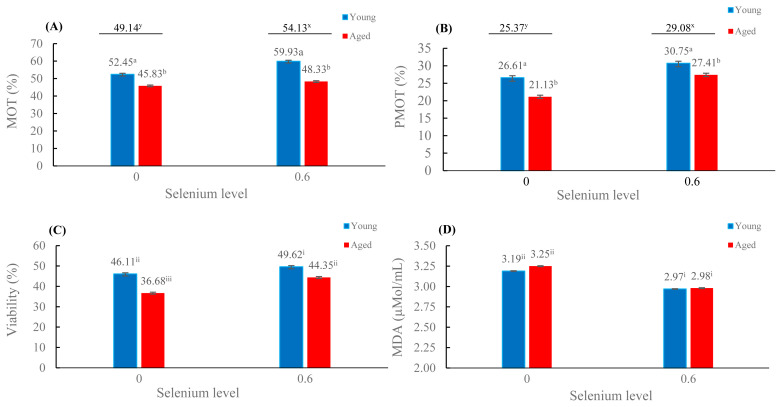
Effects of supplementation of diet with organic selenium on frozen-thawed total motility (A), progressive motility (B), sperm viability (C), and lipid peroxidation (D) in different rooster ages (mean±standard error); Values with different superscripts ^(a,b)^ are different between age groups; values with different superscripts ^(x,y)^ are different between the presence and absence of selenium groups; values with different superscripts^(i,ii,iii)^ are different between the selenium and age groups; p<0.05.

The mean percentages of total MOT and PMOT were more significant in the presence of selenium (54.13% and 29.08%, respectively) than in the absence of selenium (49.14% and 25.37%, respectively; p<0.05); these percentages were also significantly greater in young roosters than in aged roosters (p<0.05; Figure 1A–1B). The mean percentages of viability were highest in young roosters with selenium supplementation, while the lowest percentage was found in aged roosters without selenium supplementation (p<0.05; Figure 1C). Interestingly, the viability of aged roosters with selenium supplementation was comparable to that of young roosters without selenium supplementation (Figure 1C). For lipid peroxidation (Figure 1D), the mean MDA level was lower in the selenium supplementation groups than in the absence of selenium on diets (p<0.05).

Regarding fertility, as shown in Table 3, there was no interaction between age and selenium supplementation regarding fertility with an *F-value* of 0.76 (p>0.05). Younger roosters had higher fertility potential than aged roosters with an *F-value* of 126.50 (p<0.01). Likewise, dietary selenium supplementation improved fertility compared with the absence of selenium on diets with an *F-value* of 17.14 (p<0.01).

## DISCUSSION

Aging roosters typically exhibit subfertility by decreasing semen quality [10,11]; however, the factors that influence fertility reduction are poorly understood. The available data improving the fresh semen quality in aging roosters at 52 weeks of age have been provided by Akhlaghi et al [20], in which ginger extract was used, and by Adeldust et al [21], in which letrozole and herbal extract were used. A decrease in sperm abnormality and an increasing number of sperm counts were reported in those investigations, which could enhance fertility and extend the reproductive life of aged roosters. In the current study, selenium was used in the diets of young (36 weeks of age) and aging roosters (105 weeks of age) and showed that neither rooster age nor selenium in the present study affected fresh semen quality in terms of semen volume and sperm movement, even though the presence of selenium in young roosters resulted in a lower MDA content compared with aged roosters (Table 1). In aging, oxidative stress in sperm can also occur due to different factors, generating more reactive oxygen species (ROS). However, the authors assumed that those ranges in mean levels of ROS production of young and aged semen roosters would not be harmful, as they were inactivated to keep only a small amount necessary to maintain normal cell functions and not affect sperm quality [22]. The mean ranges of MDA in this study were similar to those of fresh semen chicken in a previous report [19]. Additionally, this could be confirmed by Sonseeda et al [23], who observed that age did not affect semen characteristics during a year in native Thai roosters. The difference in genetics might explain this. For example, the semen of Indian red jungle fowl could be achieved at least 4 years in the spring season [24], while semen of Thai native chickens could be collected until 79 weeks of age without affecting semen production, except during the season [11].

However, selenium supplementation in diets significantly increased sperm concentration. Accordingly, it is noted that the semen MDA concentration was significantly lower in semen with higher sperm concentration in comparison with those having low sperm counts (Table 1). This result is in accordance with previous reports in which sperm concentration was the most correlated parameter with ROS in fertile and infertile humans [25]. Selenium is required for spermatogenesis because of its vital role in the modulation of antioxidant mechanisms, which might explain why the MDA levels in this study decreased in the selenium groups. It has been suggested that testosterone concentration is positively correlated with the concentration of selenium and semen quality in several mammal species [26,27]. Moreover, in chickens, selenium plays a modulatory role by regulating the signaling pathways in chicken Sertoli cells for survival and normal functioning in the spermatogenesis process [28]. Therefore, we inferred that using selenium in diets resulted in decreased MDA levels and could improve sperm concentration in roosters. In addition, it might be suggested that in Thai native roosters, semen quality in terms of sperm concentration is mainly affected by season variations or heat stress [11], and supplementing diets with selenium might be an alternative to increase sperm counts.

In contrast with fresh semen, the younger roosters had higher post-thaw sperm quality than aged roosters (Table 2; Figure 1A–1C). However, it is of interest that the MDA concentrations after cryopreservation between different rooster ages showed similarly (Table 2; Figure 1D). This suggests that an antioxidant system that protects sperm membranes against peroxidative damage in both rooster ages would be comparable. The idea that post-thaw sperm quality declines with age might result from the structure of the sperm plasma membrane. The lipid composition of the sperm membrane impacts the resistance to cryopreservation through its contributions to membrane fluidity and stability [29]. In other words, sperm membrane fluidity behaves as an indicator of sperm freezability. A high cholesterol to phospholipids (C/PL) ratio allows the membrane to remain fluid during freezing [13]. However, the changing lipid composition with advancing age in roosters may lead to impairment in the physical and/or signal transducing properties of the sperm membranes [30], resulting in less cryotolerance of aged rooster sperm in the present study.

Regarding selenium supplementation, our findings revealed an increase in post-thaw sperm quality in terms of sperm MOT and sperm viability, while the MDA content decreased significantly (Table 2; Figure 1). This observation might be explained by the positive influence of selenium, as reported by our previous study [18]. Selenium is considered to function as an antioxidative substance that protects cells from free radicals occurring during cryopreservation. Roster sperm has high amounts of PUFAs, making sperm cells susceptible to harmful ROS [29]. Selenium protects cells in a cell culture system from oxidative damage by reducing free radical generation and inhibiting lipid peroxidation [16]. Therefore, antioxidants are needed to improve post-thaw semen. An interesting finding in the present study was that the fertility percentage increased by approximately 4% when the roosters were fed selenium (Table 3).

Very few studies look at aging and oxidative stress in the context of male reproduction in poultry. The results from this study suggest that aging is associated with an increase in ROS production; also, using selenium as dietary supplementation has the potential as an antioxidant substance. However, it might be noted that the selenium supplement level in the present study was chosen based on the previous report [18]. Therefore, it is elucidated whether a high-dose antioxidant supplementation in an aging rooster might impact semen quality. However, no study examined whether variation in either supplement over the optimal dose or longer treatment period is associated with fresh and frozen semen quality. Therefore, further investigation might be required to examine whether the higher levels of selenium supplementation can significantly improve sperm cryotolerance and fertility in the aged, comparable to those seen in young roosters.

## CONCLUSION

The present study reports that age does not influence the rooster sperm quality of fresh semen, while sperm cryotolerance and fertility were greater in young roosters than in aged roosters. However, those of aged roosters could be improved by dietary selenium supplementation. This finding is beneficial for extending the reproductive life of aged roosters and maintaining semen cryopreservation for conserving genetic diversity in aged Thai native chickens.

## Figures and Tables

**Table 1:** Comparison of fresh semen quality in the presence-absence of selenium on diets in different ages of roosters (mean±standard error)

Groups	Selenium in diets	Volume (mL)	Concentration (×10^9^ spz/mL)	Mass movement (Score 1 to 5)	MDA (250×10^6^ spz+/μMol/mL)
Young	−	0.38±0.23	4.61±1.54^y^	4.27±0.79	1.27±0.02^a,y^
	+	0.41±0.22	5.23±1.43^x^	4.38±0.61	0.85±0.02^a,x^
Aged	−	0.41±0.25	4.78±2.43^y^	4.30±0.85	1.44±0.03^b,y^
	+	0.43±0.34	5.16±2.14^x^	4.45±0.40	1.01±0.03^b,x^
Age (p-value/*F_1,20_*)		0.260/1.34	0.597/0.29	0.913/0.34	**0.001/16.35**
Selenium (p-value/*F_1,20_*)		0.428/0.65	**0.001/15.96**	0.336/2.43	**0.001/17.53**
Interaction (p-value/*F_1,20_*)		0.917/0.11	0.626/0.25	0.590/0.38	0.703/0.28

MDA, malondialdehyde.

Values with different superscripts ^(a,b)^ are different between age groups; values with different superscripts ^(x,y)^ are different between the presence and absence of selenium groups.

**Table 2:** Comparison of frozen-thawed semen quality in the presence/absence of selenium on diets in a different age of rooster

Factor	Motility parameter		Viability	MDA

	MOT	PMOT		
Age (p-value/*F_1,20_*)	**0.001/26.81**	**0.001/68.60**	0.001/131.93	0.905/1.49
Selenium (p-value/*F_1,20_*)	**0.006/9.39**	**0.001/110.96**	0.001/76.93	0.010/8.94
Interaction (p-value/*F_1,20_*)	0.205/1.72	0.236/1.49	**0.004/10.56**	**0.030/6.54**

MOT, total motility; PMOT, progressive motility; MDA, malondialdehyde.

**Table 3:** The percentages of fertility using frozen-thaw semen from the roosters (young and aged) in the presence/absence of selenium in diets (mean±standard error)

Groups	Selenium in diets	Fertility (%)	Total number of eggs
Young	−	61.94±0.69^a,y^	432
	+	64.50±0.49^a,x^	448
Aged	−	52.67±1.01^b,y^	442
	+	56.56±0.77^b,x^	470
Age (p-value/*F_1,20_*)	**0.001/126.50**		
Selenium (p-value/*F_1,20_*)	**0.001/17.14**		
Interaction (p-value/*F_1,20_*)	0.401/0.76		

Values with different superscripts ^(a,b)^ are different between age groups; values with different superscripts ^(x,y)^ are different between the presence and absence of selenium groups.
